# Integrated People-Centred Care in Canada – Policies, Standards, and Implementation Tools to Improve Outcomes

**DOI:** 10.5334/ijic.5943

**Published:** 2022-02-04

**Authors:** Patricia Sullivan-Taylor, Esther Suter, Samantha Laxton, Nelly D. Oelke, Emma Park

**Affiliations:** 1 Strategic Policy and Partner Engagement, Health Standards Organization (HSO), Canada; 2 Faculty of Social Work, University of Calgary, Canada; 3 Health Standards Organization (HSO), Canada; 4 School of Nursing, University of British Columbia, and Scientific Director, Rural Coordination Centre of British Columbia, Adjunct Faculty, Department of Community Health Sciences, Cumming School of Medicine, University of Calgary, Canada; 5 Health Standards Organization (HSO), Canada

**Keywords:** integrated care, people-centred, health systems integration, policy, standards, co-design

## Abstract

**Introduction:**

Despite the national and international policy commitment to implement integrated health systems, there is an absence of national standards that support evidence-based design, implementation, and monitoring for improvement. Health Standards Organization (HSO)’s CAN/HSO 76000:2021 – *Integrated People-Centred Health Systems* (IPCHS) National Standard of Canada (NSC) has been developed to help close this gap. This manuscript outlines the policy context and the process taken to develop the IPCHS standard.

**Description:**

The IPCHS standard is built around 10 design principles with detailed, action-oriented criteria and guidance for policy makers and health system partners. The IPCHS standard was co-designed with a technical committee that included balanced representation of policy makers, health system decision-makers, Indigenous leaders, providers, patients, caregivers, and academics. Additional feedback was received from a diverse audience during two public review periods and targeted consultation via interviews. This qualitative feedback, combined with the evidence reviews completed by the technical committee, informed the final content of the IPCHS standard.

**Discussion:**

The IPCHS standard was developed through a co-design process and complements existing frameworks by providing 66 detailed, action-oriented criteria, with specific guidance. The co-design process and consultations resulted in increased awareness and capacity among policy makers and health system partners. Supplementary tools are also in development to facilitate implementation and monitoring of progress and outcomes. This manuscript was developed in collaboration with technical committee members and HSO staff who led the targeted consultation and adoption of the IPCHS standard in six integrated care networks.

**Conclusion:**

Implementing integration strategies requires that we create and sustain a culture of continuous improvement and learning. Key lessons from the development process focused on the importance of co-design, embedding people-centred practices throughout the standard, formal yet iterative methodology inclusive of broad consultation, clear accountability for both policy makers and system partners, tools that support action and can be adapted to local context and level of integrated system maturity.

## Introduction

This analysis highlights the Canadian health policy context and evidence of the need to advance integrated people-centred health systems. Additionally, this paper demonstrates the methodology used to develop a health and social services standard through co-design with a variety of stakeholders. The aim of this standard is to facilitate integrated care policy adoption, program implementation, and evaluation.

Sustainability of a universal health care system requires transformation to be integrated and people centred. Canada’s health and social services system is a federated model with ten provincial and three territorial systems. In addition, the federal government oversees and funds the respective systems for several areas. These include the national defense, correctional services, and much of the Indigenous care system.

Canada is widely recognized for its universal health care system with national standards set out in the 1984 *Canada Health Act*, primarily covering in-hospital and physician services [1,2]. The system is funded through taxation, and services covered are then free of charge at the point of care. However, beyond the requirements of the legislation, each jurisdiction has flexibility to mandate what, if any, additional services are covered through the publicly funded system. Except for Quebec, all jurisdictions manage health and social services through separate, often multiple ministries. This further complicates the ability to coordinate services for people seeking integrated care. Like other countries, the political cycle typically limits program planning and implementation to a short window of two to four years before another election may stall, sustain, or reverse any progress [3,4,5].

Canada has an estimated population of 38 million [6], with 6.6 million seniors over 65 years of age (17.5%) [7]. In 2019, total health spending in Canada was 10.8% of GDP (Gross Domestic Product) compared to 8.8% on average in 36 Organization for Economic Co-operation and Development (OECD) countries [8]. Given additional government spending announcements in 2020 and 2021, as well as declines in some activities like elective surgeries, it is unclear where the trend will move. Early forecasting on health spending in response to COVID-19 suggests that increased costs could vary from 0.3 to 10 per cent of GDP for advanced economies, depending on whether social distancing, quarantine measures, and additional health system capacity are put in place [9].

Historically, Canada’s spending was on acute and curative care rather than preventative care and screening. In 2019, Canada spent 4.7% of GDP on curative care and 0.7% on preventative care [10]. Canada spends less on long-term continuing care and services overall than most OECD countries (in 2017, 1.3% of Canada’s GDP) [11,12].

Despite this significant spending, Canada’s population health outcomes are lagging, and many populations remain underserved. Eight OECD countries had lower health spending and higher life expectancy at birth than Canada. Canada ranked worst among 11 OECD countries for adults with lower incomes to access after-hours care without going to hospital (64% in Canada compared to 35% in the Netherlands) [13]. Over 3.2% of Canadians were waiting for treatment in 2020 and this has increased since the 1990’s [14]. One in six individuals who needed home care, including a substantial number of seniors, do not get it – and those with lower incomes, immigrants, refugees, or non-permanent residents were more likely to have unmet care needs [2].

It comes as no surprise that the integration of health and social services is a national and jurisdictional priority to address rising costs, changing population needs, long waits, limited capacity of hospital, and long-term care beds and inequity [11,12,15]. “To fully achieve the potential of Canada’s Medicare system, action on the social determinants of health…must occur in parallel with health system reform. Without bold political vision and courage to strengthen and expand the country’s health system, the Canadian version of universal health coverage is at risk of becoming outdated” [16].

Integration exists in all strategic direction documents from governments, health authorities, and health care organizations. This was further reflected in the recent *Canadian Quality and Patient Safety Framework*, which highlights integrated care as one of five areas to target improvement efforts. The framework has been endorsed by the federal government and several provincial governments [17] and is being used to inform HSO’s quality and patient safety products and services.

COVID-19 exacerbated gaps for equitable care and made visible the chasms that exist for vulnerable populations that are most likely to benefit from integrated care [18,19]. These include people with disabilities, Indigenous people, people in need of housing or food, the LGBTQ2I community, the elderly, and people in correctional facilities [20]. The pandemic accelerated the need for integration and coordination of health and social services. Fragmentation in health and social care systems inhibited the quick, collaborative, and large-scale reactions needed to effectively respond [21,22,23].

Despite the national and international commitment to implement integrated delivery systems, there is an absence of national standards that support evidence-based design, implementation, and monitoring for improvement. Interviews with clients and other stakeholders concluded that much of the work to date has focused on frameworks that they perceived as theoretical, too high-level to support integrated health system design, implementation, and evaluation. The results of consultation signalled a clear need for a standard that would help users to operationalize the evidence in a variety of settings and populations, with clear direction for what to do and who was most accountable.

Health Standard Organization’s (HSO) CAN/HSO 76000:2021 – Integrated People-Centred Health Systems (IPCHS) standard has been developed to help close this gap. The IPCHS standard is for use by global health and social service ministries, administrators, and authorities as well as system provider organizations and networks that have identified the integration of health and social services as a priority. The standard is divided into 10 design principles that address various aspects of health and social service integration. Each design principle is supported with action-oriented criteria and guidelines that outline preliminary requirements for effective health system integration [24].

*HSO* and *Accreditation Canada (AC)* are Canadian affiliated, not-for-profit organizations dedicated to improving the quality of health and social services around the world. Over the past two years, HSO responded to client input and their calls for guidance on how to deliver integrated people-centred health systems.

## Background and Context

Over the past several years, Canadian jurisdictions have introduced policies and programs to advance more integrated care. There are numerous integrated care initiatives for specific populations (e.g., frail seniors) or targeted conditions (e.g., mental health and addictions, stroke). However, the scope of this manuscript is on system-level policy examples that have been implemented federally and in the provinces of British Columbia (BC), Saskatchewan (SK), Ontario (ON), Quebec (QC) and Nova Scotia (NS). The Canadian federated health and social services model means that implementation of the IPCHS standard is more likely to be advanced through a provincial or territorial effort. Consequently, it was important to understand policy initiatives at this level. Furthermore, these policy initiatives influenced the IPCHS standard content and will impact its adoption in design, implementation, and evaluation of integrated care systems.

In 2019, Health Canada funded a three-year pan-Canadian program to build HSO standards and implementation tools that would improve the integration of youth mental health and addiction services. These resources are co-designed with youth, families, providers, community members and policy makers and tested in community settings to learn how they drive and sustain change [25].

The First Nations Health Authority (FNHA) in BC designs, manages, and funds the delivery of First Nations health programs and services on behalf of 203 diverse First Nations communities [26]. This involves collaboration with the BC Ministry of Health and BC’s six health authorities. FNHA’s mandate is to coordinate and integrate the respective health programs and services to achieve better health outcomes for First Nations in BC [27]. The FNHA programs aim to be integrated with the BC health system through partnerships with provincial and federal governments, service providers and BC First Nations communities. Furthermore, the BC First Nations Perspective on Health and Wellness is embedded in the health care system, shifting from a sickness-treatment model into a wellness model. They also have a specific objective to promote cultural safety and humility in the health care system [28].

The Saskatchewan Ministry of Health reinforced their commitment toward more integrated people-centred care, beginning in 2011, with the release and implementation of the *Patient-and Family-Centred Care (PFCC) Framework*. The framework has guided adoption of PFCC as the foundation of the health system [29]. It also produced the Patient’s Medical Home, a new vision for integrated primary care that puts patients and their families at the centre. This team-based primary care model has since been adopted across Canada [30]. A 2016 Health System Report followed, which recommended consolidating 12 Regional Health Authorities into a single provincial health authority “focused on meeting patient needs through seamless, integrated and team-based care” [31]. The transition to a single health authority occurred in 2017 and in fall 2019, SHA met accreditation requirements as a unified health system [32]. Their continued focus is on connected care and improved system-wide coordination and alignment [33].

Ontario initiated various integrated care models for more than ten years. Its most recent large-scale system transformation was the introduction of 24 Ontario Health Teams (OHTs) in 2019. This included a combination of bottom-up and top-down actions – such as the implementation of Bill 74, *The People’s Health Care Act*, 2019; intended to advance integrated care [34]. By fall 2020, a total of 42 OHTs and an investment of $28 million in fiscal year 2020–2021 had been announced [35]. The aim of OHTs is to end hospital overcrowding, reduce wait times and improve outcomes while building a “connected and sustainable public health care system centred around the needs of patients”. The OHTs must ensure 24/7 navigation and care coordination for patients and families – providing a seamless experience between health care services, providers, and settings [36]. As in other international integrated delivery models [4], the bottom-up action allows each OHT to determine their local population needs and areas of service priority. This informed the necessary partnerships and associated measurable outcomes. Through the early evolution of OHTs, debate has included the use of standards versus standardized practice to promote innovation and reduce unwarranted care variation [37].

Likewise, Quebec introduced legislation in 2015 that established Integrated Health and Social Services Centres and Integrated University Health and Social Services Centres [38]. The legislation consolidated governance to centralize decision making and achieve more community-oriented integrated care [39]. It was intended to make it easier to assure the continuity of care for those needing more than one point of care. Concurrently, the government worked with physician practices to facilitate more team-based group practice models. Although this increased overall population access to primary care, there continues to be persistent inequities of access for those most vulnerable. In fact, the pandemic has reinforced the need for strengthened links between public health and primary care [23]. In 2017, the Quebec Ministry of Health and Social Services mandated accreditation. They worked with AC to co-design an assessment program that shifted the focus of accountability for quality from within organizations toward the system that clients and their families experience [40]. Concurrently, the government worked with physician practices to facilitate more team-based group practice models and promoted interdisciplinary care.

In 2015, the Nova Scotia government amalgamated nine district health authorities to form the Nova Scotia Health Authority (NSHA) [41]. The goals were to increase access, enhance the safety and quality of care, and make the best use of resources [42]. There has been progress on reducing wait times and improving patient flow (e.g., mental health and addictions, emergency, and inpatient care) [43,44]. NSHA also introduced integrated care programs for specific populations, including chronic care, palliative care, and gastrointestinal disorders. These programs focus on the patients’ goals and include comprehensive assessments, coordinated care planning with individuals and their interdisciplinary team and care delivered in the most appropriate setting [45,46,47].

This analysis highlights the Canadian policy context and ongoing efforts to advance integrated people-centred health systems. While developed in Canada, the IPCHS standard, supplementary implementation tools, and learnings are global resources designed to facilitate integrated care policy and program implementation and evaluation. The subsequent sections highlight the IPCHS standard development methodology and core content, the international evidence and context and the lessons that can be applied elsewhere.

## Methodology

The IPCHS standard outlines evidence-informed requirements to design, implement, and evaluate integrated health and social service systems. It is targeted to policy makers and system partners that are responsible for funding, planning, coordinating, delivering, and evaluating integrated health and social services in Canada and internationally [24].

### IPCHS Standard Development Process

**Figure 1:**
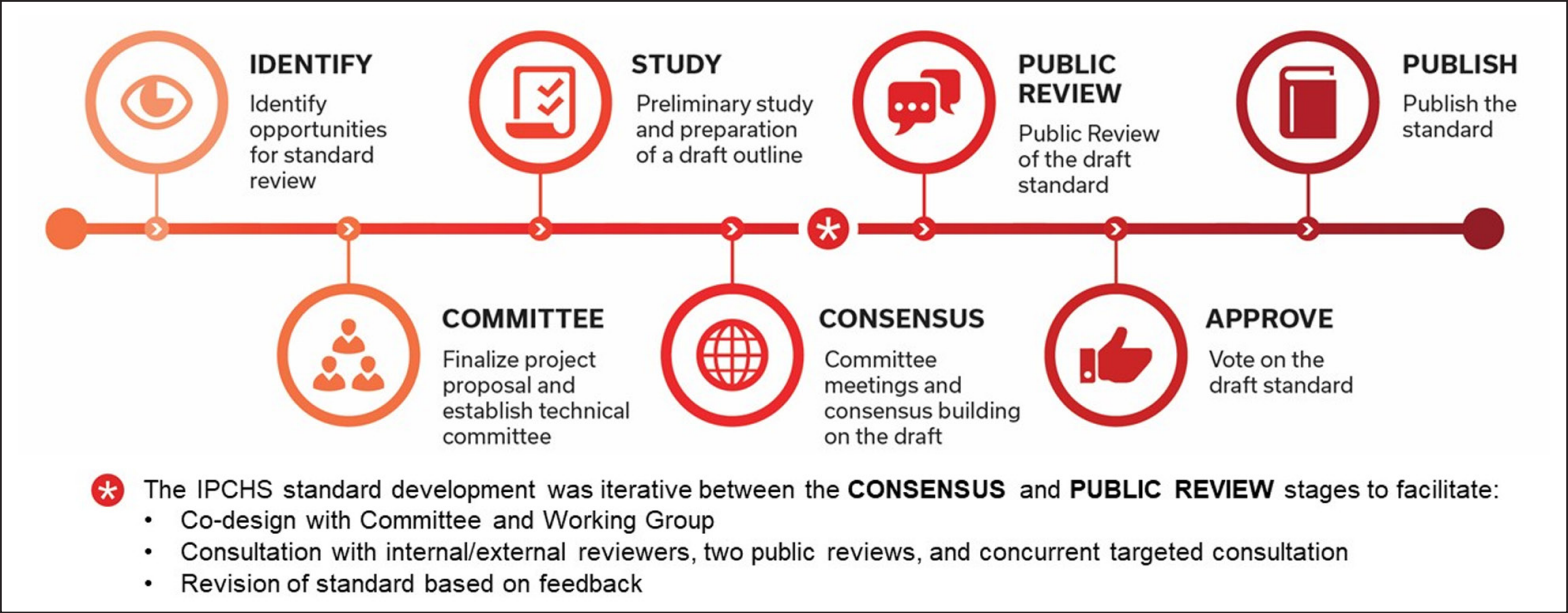
Methodology and timeline for co-designing the Integrated People-Centred Health Systems Standard (HSO 2021).

The standard was developed following the HSO accredited standards development process (see ***Figure 1***) with adaptations to allow for the complexity and expressed needs of multiple stakeholders. The technical committee that developed the standard consisted of balanced geographic and lived-experience representation including policy makers, health system decision-makers, Indigenous leaders, providers, patients, caregivers, and academics. During the study phase, the team conducted a comprehensive review of national and international literature on people-centred integrated care models and factors impacting the quality and safety of care. The evidence was updated throughout the standard development process. Concurrent to the public reviews, there was targeted consultation through in-person and virtual interviews with 80 health care leaders, health system partners and policy makers. The aim was to test the clarity, relevance, and usability of the standard. The qualitative feedback gathered through public review and interviews combined with the evidence reviews informed the final content of the IPCHS standard. The consultation also resulted in increased awareness and capacity among policy makers and health system partners. The technical committee reviewed more than 600 comments in order finalize the IPCHS standard.

Based on the systematic literature review, we chose to lean on the *WHO global framework* [48] to conceptualize the standard. The draft standard was organized into the following subsections:

- Planning and designing people-centred system,
- Creating and sustaining an enabling environment,
- Allocating resources and building infrastructure, and
- Supporting coordinated and comprehensive care.

The standard was based on the following definition, adapted from the WHO [49].

#### Integrated people-centred health and social services

*Health and social services that are organized and managed across sectors and organizational boundaries, so people and communities receive coordinated and comprehensive services that address the social determinants of health and meet the health and well-being needs of people and communities from birth to end of life*.

There were several core principles that underpinned the draft standard: person, family, and community-centred; coordinated and continuous, comprehensive, governed through shared accountability, evidence-informed, and ethical.

Partners were engaged through regular meetings on the technical committee and offline contributions in areas of expertise. Work was conducted over two years to build consensus and develop a set of comprehensive criteria and guidelines under each subsection to facilitate implementation.

**Figure 2:**
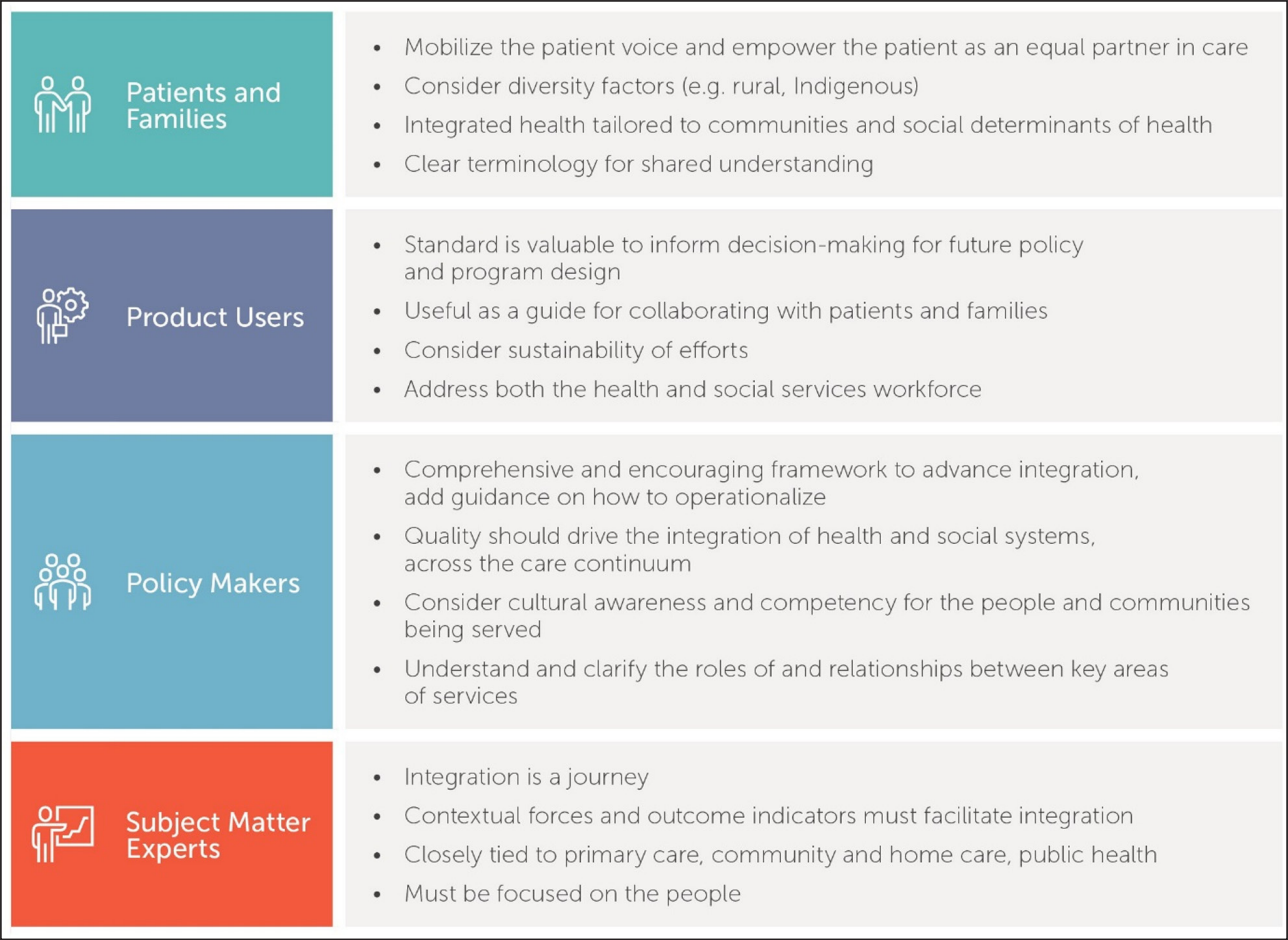
Summary of stakeholder input from initial IPCHS standard public review (HSO 2018).

Feedback from the first public review was substantive and represented a critical juncture in the standard development (see ***Figure 2***).

**Figure 3:**
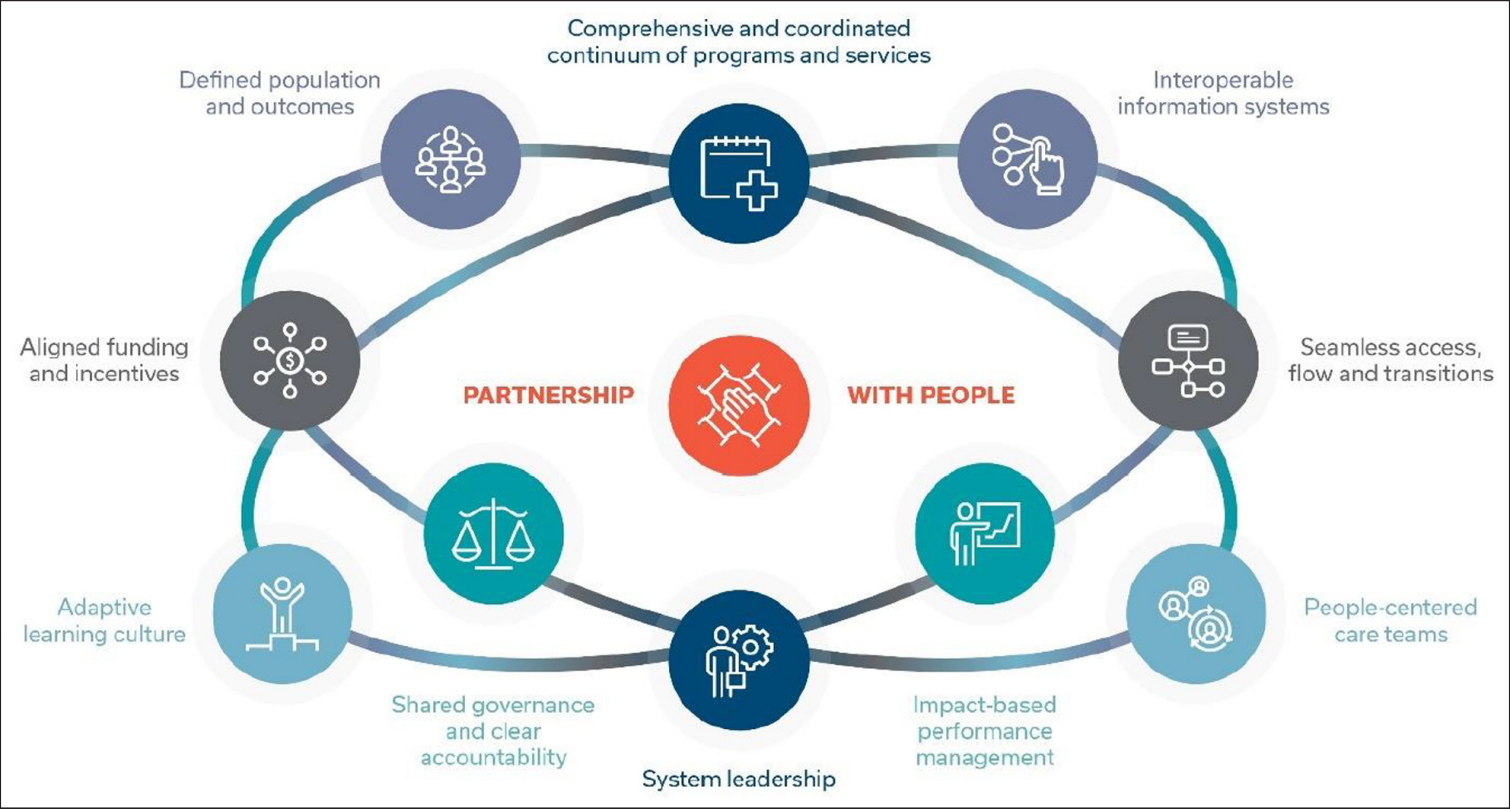
Ten design principles for Integrated People-Centred Health Systems (HSO 2021).

This public feedback led to a significant reconceptualization and restructuring of the standard to include:

- An adapted and refined version of the 10 key principles for integrated health systems [50] with content organized around each principle to improve clarity. Principles were supplemented with recent evidence to reflect the evolving understanding of integrated care (see ***Figure 3***) [24].
- Separated guidelines into macro (policy) and meso (operational) directives to reflect governance, accountability, and responsibility to increase adoption and operationalization.
- Identified gaps and added criteria (where applicable) from the European Network Reference framework [51].
- Developed a comprehensive glossary to create a shared understanding of terms and concepts used in the standard.

Revising the standard under the new design principles and separating guidelines for policy and health system operations took considerable time. ***Table 1***. highlights the main components included in each design principle. To make the standard relevant and actionable in the real world each design principle has a set of criteria that set out explicit, objective requirements to be met. The standard has 66 criteria in total that people can use as aspirational targets to meet in their journey toward a fully integrated health system. Guidance is available for each principle as well, to help policy makers and system partner organizations know what actions to take as they set and advance their own local priorities. The actionable criteria allow for variation in local context, populations and scale of maturity to ensure the standard can be applied universally.

**Table 1:** Ten Design Principles and Associated Components within the Integrated People-Centred Health Systems Standard (HSO 2021).


PRINCIPLE	COMPONENT

1. Define the population and identify desired health and well-being outcomes	Define the population served Define health and well-being outcomes Partner with people and communities Conduct population-based needs assessments

2. Coordinate a comprehensive continuum of services	Collaborate to design comprehensive and coordinated programs and services Include all services required to achieve the defined population’s desired health and well-being outcomes throughout the life course Coordinate care and services within and across health systems

3. Optimize access, flow, and transitions	Improve the experience of the people using and delivering services Design services that are timely, equitable, and physically, emotionally, economically, and culturally accessible Enhance access to primary health care Develop, support, and maintain processes that facilitate service transitions

4. Enable and support people-centred care teams	Use people-centred teams to deliver services Establish and maintain competencies to deliver team-based care and services Include people and communities as members of the care teams Evaluate team functioning

5. Develop and strengthen system leadership	Collaborate with people and communities to design a vision for health system integration Establish an evidence-informed leadership model Develop and strengthen leadership skills

6. Establish shared governance and clear accountabilities	Establish a shared governance and clear accountability structure Include people and communities, and relevant sectors, in shared governance structures Formalize accountability arrangements

7. Align funding and incentives	Establish a value-based health care approach Implement and evaluate payment, remuneration, and financial and non-financial incentives Implement and evaluate integrated health and social service budgets Recognize and reward collaborative and integrated behaviours Share responsibility for financial sustainability

8. Implement interoperable information systems	Identify a shared data and information governance framework Establish and maintain interconnected and interoperable digital and information technology environments Develop and maintain the policies, procedures, and infrastructure required throughout the information lifecycle

9. Measure and manage performance based on impact	Design and implement a performance management framework Collaborate with people and communities, providers, and sectors to identify performance outcomes Implement continuous quality improvement activities and initiatives

10. Embed an adaptive learning culture	Build an adaptive and dynamic learning culture Share innovation from the top down and bottom up Participate in knowledge-sharing activities Encourage learning and knowledge translation Involve people and communities in the learning culture


The draft standard underwent several review cycles by the technical committee to reach consensus and consistency on the new structure and guidelines. Given the substantive revisions made to the initial draft standard, there was a second public review. At this point, there was strong endorsement for the standard and revisions were mostly editorial.

## Discussion – Canadian and International Context and Relevance

Health and social service integration is a continuous process of quality improvement. It requires trust, partnerships, and collective action toward a common goal between policy makers, system partners, clients, and their families. Shared policies, procedures and practices across health and social services need to be developed to address the social determinants of health and improve population health outcomes.

We have much to learn on how to implement, monitor and sustain strategies and approaches to achieve integrated health systems. Barriers often include policy and legislation (e.g., political cycles, fragmented funding models), lack of governance and accountability structures, disconnected information systems, and lack of workforce competencies [52,53].

Over the last decade, several models and frameworks have emerged to support jurisdictions in their integration journeys [54]. Some of these, such as the SCIROCCO framework [55,56], the Project Integrate framework [57], the Development Model for Integrated Care (DMIC) [58] and the Rainbow Model for Integrated Care (RMIC) [59] facilitate the planning, implementation, and/or assessment of integration services. In contrast, the Context, Outcomes, and Mechanisms of Integrated Care (COMIC) model [60] and the Integrated Care Performance Assessment (ICPA) framework [61] are more focused on outcomes or performance of integrated care systems. These multilevel conceptual frameworks shine light on the complexity and multilayered relationships emerging in health systems.

The IPCHS standard complements these frameworks by providing a comprehensive quality improvement tool. This standard guides policy makers and system partners on what to do, as well as how to engage with people and communities. What makes the IPCHS standard unique from other frameworks is the way it provides 66 detailed, action-oriented criteria, with specific guidance. This information allows a health system, network or jurisdiction to co-design, implement, and evaluate the structures, policies, strategies and plans to reach and sustain targeted integration goals. The Canadian policy examples demonstrated that integration takes many forms – from local health teams and new primary care models to integration of geographic health authorities into unified entities. Irrespective of the level and scope of integration, there are universal principles to be considered in the planning and implementation. The IPCHS standard captures these principles and offers specific guidelines for policy makers and health system partners.

The standard criteria are aspirational, acknowledging that integration is a journey. If consistently applied, the standard will enable transformation towards systems that are people-centred and co-created by the communities they serve. Partnerships and engagement were key elements in the development of the standard. The inclusive approach to stakeholder consultations meant that a variety of voices helped shape the broader vision as well as the specific criteria. This ensured the standard contains the integration building blocks that truly matter to people.

*“The IPCHS standard focuses on expanding boundaries and partnership…inclusive of not only health and social services but on health and well-being as identified by community stakeholders. It moves away from institutional-based care to community-based care requiring health care systems to collaborate, communicate, and co-create beyond their borders.”*

*Patient Partner and HSO IPCHS Standard Technical Committee Member*

The track record of HSO as an authority dedicated to health and social services standards and assessment programs lent credibility to the work and facilitated broad engagement. HSO’s standards are implemented in more than 15,000 locations globally, in 38 countries, including all 13 Canadian provinces and territories. These standards and assessment programs are used by accrediting bodies to provide quality and safety assessments and recognition programs for health and social services [62]. The reputation and broad reach of the organization is expected to foster general acceptance and quicker adoption of the IPCHS standard.

While the release of the standard is an important milestone, it is just the beginning. Many are at initial stages of their integration journey. Consequently, now is the time to build understanding, knowledge and capacity among policy makers, the workforce, individuals, and communities on what integrated people-centred health systems mean within their context. This manuscript was developed in collaboration with Technical Committee members and HSO staff who led the consultation and adoption of the IPCHS standard in six integrated care networks.

The IPCHS design principles and associated criteria support education and training programs to align language and build competencies and capacity to enable integrated systems. For example, *PAQS*, a system quality improvement organization in Brussels, Belgium, is implementing the standard through a series of educational workshops. The aim is to build a shared definition and understanding within the workforce of integrated care and the role each person plays to achieve integration goals [63]. Awareness building is a foundational component to the adoption of best practices and implementation of integrated, people-centred health systems.

*“As we are committed to accreditation, this is a way to adjust standards we already use at the meso and operational levels. For me, at the Board level, it won’t be difficult to adopt.”*

*Nurse, Board Member – Integrated University Health and Social Services Centre, and HSO IPCHS standard Technical Committee Member*

The Canadian Quality and Patient Safety Framework highlights the need for practical tools to facilitate implementation and monitoring of progress and outcomes. To address this need, HSO is working with integrated youth networks and other content experts to co-design and implement an Integrated Care Pathways Toolkit. This tool is different from the IPCHS standard, which addresses the macro and meso levels of integration. Rather, the Pathways Toolkit is meant to be used at the direct patient/provider care level. Integrated care pathways are locally defined solutions that connect people to the right services across the continuum of care. Integrated care pathways allow for equitable access to services that are safe (physically, psychologically, culturally), people-centred and coordinated among the care teams. The Pathways Design Tool will build upon existing capacity and enablers, such as care navigators/coordinators and interdisciplinary care teams.

This integrated care implementation tool aims to serve as a guide for how a health and social service network can collaborate with people and communities to understand the current care experience and health outcomes for a defined population. The tool will also help teams to co-design a vision for the desired integrated care pathway. HSO is piloting its Pathways Toolkit in 2021 and 2022 with a final release expected in late 2022.

Monitoring progress towards integrated health and social services systems as well as the outcomes of integrated systems remains a challenge [64, 54]. The OECD is working with member countries to benchmark progress of integrated care delivery via *new indicators* and mapping of integrated care policies [65,66]. Consistent monitoring enables the identification of pockets of excellence that should be celebrated and potentially scaled, as well as areas where improvements are needed. Understanding progress depends on the readiness and capacity to monitor performance and a shared accountability for improvement across policy makers, system partners, clients, and their families.

Although the IPCHS standard sets out criteria for monitoring, it does not assume all systems will have the capacity to do so. Monitoring requires the appropriate workforce, tools, and infrastructure to compare progress and performance across systems and time. To support near-term monitoring and the IPCHS standard implementation, work is underway on an *Integrated Care Assessment Tool* that is directly aligned to the design principles and criteria. This tool is for use by health system partners and policy makers who wish to evaluate their current state and advance progress toward their integration goals. The tool will be piloted in six integrated youth service networks across Canada in 2021 and 2022. A final Integrated Care Assessment Tool is expected to be released in late 2022 that incorporates feedback from the pilot.

## Lessons Learned and Conclusion

Integrated health systems may vary in their structures. However, integrated health systems must be designed to improve population health and quality of care, create meaningful engagement within care teams, and provide value to people and communities [18,67]. Specific approaches for meeting integration requirements will vary. Tactics differ depending on local context, jurisdictional laws and regulations, and populations served. Ultimately, implementing integration strategies requires that we create and sustain a culture of continuous improvement and behaviour as a collective learning health system.

The policy agenda in Canada and internationally is clear. That said, delivering and sustaining an integrated people-centred health system requires a suite of tools that align and support policy implementation and evaluation. The IPCHS standard complements existing frameworks while providing the additional granularity stakeholders are seeking. The standard and supplementary assessment and pathways design tools will advance integrated people-centred health system planning, implementation, and evaluation.

Key learnings from the standard development focused on the importance of co-design, embedding people-centred practices throughout the standard, formal yet iterative methodology, clear accountability for both policy makers and system partners, tools that can be adapted to local context and level of integrated system maturity.

- Co-design in partnership with a balanced group of stakeholders was essential to create a standard that has utility and is accepted by diverse user groups.
- People-centredness is embedded throughout the standard (rather than limited to a single design element) to ensure that the individual and populations served are at the center of all integration activities.
- Formal methodology with iterative design enabled ongoing consultation and several rounds of multi-stakeholder feedback. This increased awareness and capacity among policy makers and health system partners.
- The standard sets clear expectations and accountabilities for policy makers and system partners; reinforcing that integrated care implementation will require strong partnership.
- The standard is flexible to accommodate local context and use by networks and/or health systems regardless of what stage they are at in the integration journey. For those in the early phases of integration, health system partners and policy makers can use the standard to inform strategic planning and funding decisions. Where integration efforts are more mature, the standard will help to evaluate and advance efforts.
- The standard combines a top-down and bottom-up approach by outlining overarching and clear criteria on what integration looks like – while allowing local integration processes to unfold.
- Creating fully integrated people-centred health systems is a complex undertaking that takes years to achieve. Consequently, accreditation is not warranted at this early stage. As an alternate, no replacement text tools such as the Integrated Care Assessment Tool, will reinforce the value and use of the standard.
